# Geriatric Patient Safety Indicators Based on Linked Administrative Health Data to Assess Anticoagulant-Related Thromboembolic and Hemorrhagic Adverse Events in Older Inpatients: A Study Proposal

**DOI:** 10.2196/resprot.7562

**Published:** 2017-05-11

**Authors:** Marie-Annick Le Pogam, Catherine Quantin, Oliver Reich, Philippe Tuppin, Anne Fagot-Campagna, Fred Paccaud, Isabelle Peytremann-Bridevaux, Bernard Burnand

**Affiliations:** ^1^ Institute of Social and Preventive Medicine Lausanne University Hospital Lausanne Switzerland; ^2^ Faculty of Biology and Medicine University of Lausanne Lausanne Switzerland; ^3^ Biostatistics and Bioinformatics (DIM) Dijon University Hospital and University of Bourgogne Franche-Comté Dijon France; ^4^ Inserm, CIC 1432 Clinical epidemiology / clinical trials unit Dijon University Hospital Dijon France; ^5^ Inserm, UMR 1181, B2PHI: Biostatistics, Biomathematics, PHarmacoepidemiology and Infectious diseases Institut Pasteur and Université de Versailles St-Quentin-en-Yvelines, Université Paris-Saclay Paris France; ^6^ Department of Health Sciences Helsana Insurance Group Zürich Switzerland; ^7^ Caisse Nationale d’Assurance Maladie des Travailleurs Salariés Paris France

**Keywords:** patient safety indicators, older inpatients, acute care hospital, anticoagulant-related adverse events, adverse drug events, administrative health data, hospital discharge data, insurance claims data, linked data

## Abstract

**Background:**

Frail older people with multiple interacting conditions, polypharmacy, and complex care needs are particularly exposed to health care-related adverse events. Among these, anticoagulant-related thromboembolic and hemorrhagic events are particularly frequent and serious in older inpatients. The growing use of anticoagulants in this population and their substantial risk of toxicity and inefficacy have therefore become an important patient safety and public health concern worldwide. Anticoagulant-related adverse events and the quality of anticoagulation management should thus be routinely assessed to improve patient safety in vulnerable older inpatients.

**Objective:**

This project aims to develop and validate a set of outcome and process indicators based on linked administrative health data (ie, insurance claims data linked to hospital discharge data) assessing older inpatient safety related to anticoagulation in both Switzerland and France, and enabling comparisons across time and among hospitals, health territories, and countries. Geriatric patient safety indicators (GPSIs) will assess anticoagulant-related adverse events. Geriatric quality indicators (GQIs) will evaluate the management of anticoagulants for the prevention and treatment of arterial or venous thromboembolism in older inpatients.

**Methods:**

GPSIs will measure cumulative incidences of thromboembolic and bleeding adverse events based on hospital discharge data linked to insurance claims data. Using linked administrative health data will improve GPSI risk adjustment on patients’ conditions that are present at admission and will capture in-hospital and postdischarge adverse events. GQIs will estimate the proportion of index hospital stays resulting in recommended anticoagulation at discharge and up to various time frames based on the same electronic health data. The GPSI and GQI development and validation process will comprise 6 stages: (1) selection and specification of candidate indicators, (2) definition of administrative data-based algorithms, (3) empirical measurement of indicators using linked administrative health data, (4) validation of indicators, (5) analyses of geographic and temporal variations for reliable and valid indicators, and (6) data visualization.

**Results:**

Study populations will consist of 166,670 Swiss and 5,902,037 French residents aged 65 years and older admitted to an acute care hospital at least once during the 2012-2014 period and insured for at least 1 year before admission and 1 year after discharge. We will extract Swiss data from the Helsana Group data warehouse and French data from the national health insurance information system (SNIIR-AM). The study has been approved by Swiss and French ethics committees and regulatory organizations for data protection.

**Conclusions:**

Validated GPSIs and GQIs should help support and drive quality and safety improvement in older inpatients, inform health care stakeholders, and enable international comparisons. We discuss several limitations relating to the representativeness of study populations, accuracy of administrative health data, methods used for GPSI criterion validity assessment, and potential confounding bias in comparisons based on GQIs, and we address these limitations to strengthen study feasibility and validity.

## Introduction

### Background

People aged 65 years and over are the most frequent users of acute care hospitals in Europe, and aging trends among hospital inpatients are expected to increase dramatically in the next decades [1,2]. However, acute care hospitals often deliver substandard care to older people with complex care needs [3]. Moreover, frailty, chronic multimorbidity, disability, polypharmacy, and the resulting clinical and organizational complexity of care [4] expose older inpatients to an increased risk of hospital care-related adverse events [5-7]. A literature review not only confirmed the high incidence of adverse events among older inpatients, accounting for 5% to 60% of admissions in acute care hospitals, but also highlighted the strong association between adverse events and hospital care quality, with more than 50% of these events being deemed preventable [7]. These adverse events have important consequences for older patients, as they accelerate the aging process and lead to loss of autonomy, frequent and longer hospitalization, institutionalization, and finally death [4,6,7]. Adverse events also worsen patients’ experience with hospital care and affect their quality of life [4,8]. Finally, they weigh on health services utilization and costs [4,6].

Like most countries, Switzerland and France have initiated systemic reforms to move toward a more sustainable health care system and meet the challenges of aging and chronic multimorbidity [9]. Both governments give priority to health care quality improvement in older patients and foster the provision of better data to inform health policy, promote transparency, and improve health care efficiency [10-15]. Indeed, quality and safety indicators targeting older inpatients are essential to support and drive quality improvement, as well as inform health care stakeholders. These indicators are also of great interest to compare the performance of various health systems [16]. Some commonly used indicators based on large administrative health databases (eg, hospital discharge and insurance claims databases) could help assess and compare patient safety and health care quality across hospitals and health territories in both countries. They could also enable comparisons of Swiss and French health systems’ performance in providing high-quality, safe care to vulnerable older inpatients [12,15], which are a source of “cross-country learning” and improvement [17,18]. For example, the Patient Safety Indicators (PSIs) [19], which have been developed by the US Agency for Healthcare Research and Quality (AHRQ) [19] and adopted internationally [20,21], could screen acute hospital discharge data for potentially avoidable adverse events occurring during hospitalization. Similarly, PSIs adapted to linked administrative health data (ie, hospital discharge data linked to insurance claims data) could be of use to monitor in-hospital and postdischarge adverse events, as the latter may reflect delayed and poor quality of hospital care or premature discharge from hospital [22]. Finally, some of the Assessing Care of Vulnerable Elders-3 (ACOVE-3) quality indicators could evaluate health care processes and medication management in older inpatients based on administrative data [3,23].

Among adverse events affecting older inpatients, anticoagulant-related thromboembolic and hemorrhagic adverse events are especially frequent and serious; in fact, age is one of the strongest predictors of venous or arterial thromboembolism and bleeding during anticoagulation [24-26]. Furthermore, thromboprophylaxis is frequently suboptimal in older inpatients despite the availability of professional guidelines [27]; many studies have indeed reported recurrent prescriptions of supratherapeutic doses of anticoagulants, as well as frequent underuse and rare risk-benefit assessment of anticoagulation in this population [26-30]. Finally, the growing use of anticoagulants, especially of direct oral anticoagulants, in the geriatric inpatient population and their substantial risk of toxicity and inefficacy have become an important patient safety and public health concern worldwide [25,31]. Anticoagulant-related adverse events and the quality of anticoagulation management should therefore be routinely assessed in older inpatients receiving anticoagulants for the prevention and treatment of arterial or venous thromboembolism.

Two PSIs can be used to monitor potentially avoidable perioperative thromboembolic or bleeding events in older Swiss and French inpatients: PSI-12, Perioperative Pulmonary Embolism or Deep Vein Thrombosis Rate, and PSI-09, Perioperative Hemorrhage or Hematoma Rate [19,20,32-34]. Indeed, although both indicators target adult inpatients, most corresponding adverse events affect inpatients aged 65 years and over [20]. Moreover, PSI-12 has been adapted to coding systems of various countries, including Switzerland and France [21,35]. Lastly, both algorithms have been extended to capture both in-hospital and postdischarge adverse events based on hospital discharge data linked to outpatient claims data [22]. Besides these perioperative PSIs, others should be developed to monitor adverse events related to venous thromboembolism curative treatments and thromboprophylaxis in at-risk medical conditions such as severe acute infection or atrial fibrillation. Case-mix adjustment of PSIs should also be considered to account for differences in older inpatients’ clinical risks or disease severity at admission [4] and enable comparisons across hospitals or geographic areas [36,37]. In particular, PSIs should be adjusted for older inpatients’ risk factors for both hospital care-related adverse events (eg, frailty, chronic multimorbidity, disability, or polypharmacy) and thromboembolic or hemorrhagic adverse events (eg, age ≥75 years, renal or liver failure, inherited or acquired disorders of hemostasis, malignancy). Thus, besides the Charlson comorbidity index [38] and the updated chronic disease score [39], which have already been adapted to Swiss and French administrative health data, other comorbidity indexes [40], proxy measures of frailty and disability [41,42], and individual risk scores of thromboembolism or hemorrhage [43,44] should also be developed or adapted [45].

Regarding process quality metrics, three ACOVE-3 indicators may be used to evaluate warfarin prescription and surveillance in older patients with heart failure or atrial fibrillation [23]. Additional indicators should be developed to assess the management of other anticoagulants, including direct oral anticoagulants.

### Objectives

This research project aims to develop and validate a set of outcome and process indicators based on linked administrative health data (ie, insurance claims data linked to hospital discharge data) assessing older inpatient safety related to anticoagulation in both Switzerland and France, and enabling comparisons across time and among hospitals, health territories, and countries.

The project will thus comprise complementary steps aiming to (1) develop and validate a set of geriatric patient safety indicators (GPSIs) assessing in-hospital and postdischarge anticoagulant-related adverse events in older inpatients; (2) adapt the PSI-09 and PSI-12 to Swiss and French linked data; (3) develop a set of geriatric quality indicators (GQIs) assessing the management of anticoagulants for the prevention and treatment of arterial or venous thromboembolism based on Swiss and French data; (4) develop or adapt chronic multimorbidity indexes, proxy measures of frailty and disability, and thromboembolic and bleeding risk scores based on Swiss and French data to adjust GPSIs on case mix; and (5) compare anticoagulation safety within and between Switzerland and France.

## Methods

### Development and Validation Process

**Figure 1:**
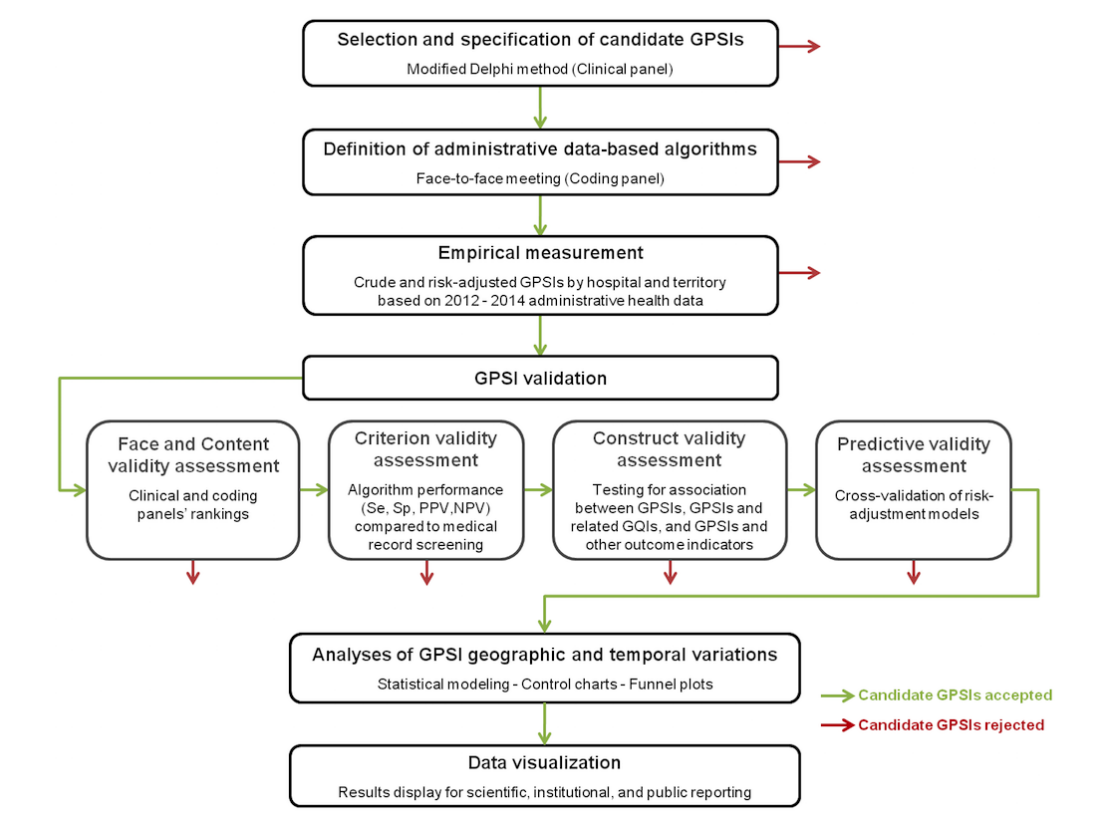
Summary of the geriatric patient safety indicator (GPSI) development and validation process. GQI: geriatric quality indicator; NPV: negative predictive value; PPV: positive predictive value; Se: sensitivity; Sp: specificity.

We will develop and validate GPSIs according to the methodology used by the AHRQ [46-48], which comprises 6 standardized sequential stages (Figure 1).

#### Selection and Specification of Candidate GPSIs

We will use a modified Delphi method [49] combining evidence from a systematic literature review with the collective judgment of clinical experts (clinical panel) to select candidate indicators and define their specifications (numerator, denominator, risk-adjustment factors, and measurement time frame for postdischarge adverse events). These candidate GPSIs will measure the cumulative incidences of thromboembolic and hemorrhagic adverse events for selected surgical procedures or medical conditions (Textbox 1).

**Candidate geriatric patient safety indicators (GPSIs) and geriatric quality indicators (GQIs) assessing older inpatient safety regarding anticoagulation.**

**GPSIs**

Cumulative incidence of in-hospital and postdischarge anticoagulant-related adverse events:

1. Venous thromboembolism or hemorrhagic events in surgical patients

- High-risk surgery (eg, total hip or knee arthroplasty)
- Moderate-risk surgery (eg, abdominal and pelvic surgery)
- Low-risk endoscopic surgery or diagnostic procedures (eg, colonoscopy)

2. Venous thromboembolism or hemorrhagic events in medical patients

- Acute medical conditions (eg, severe acute infection, acute heart failure, nonsurgical trauma)
- Chronic conditions (eg, cancer, chronic inflammatory diseases)

3. Strokes and other systemic arterial embolisms or hemorrhagic events in patients with atrial fibrillation

4. Recurrent venous thromboembolism or hemorrhagic events in patients with venous thromboembolism

5. Adapted Patient Safety Indicator (PSI) -12 (perioperative thromboembolism)

6. Adapted PSI-09 (perioperative hemorrhagic event)

**GQIs**

For each GPSI, 2 GQIs assessing the management of anticoagulant treatments:

1. Proportion of index hospital stays resulting in the recommended anticoagulation (drug, dose, frequency, duration) at and after discharge

2. Median duration of anticoagulant treatment after discharge for index hospital stays

#### Definition of Administrative Data-Based Algorithms

GPSI algorithms will be determined by Swiss and French administrative health data experts (coding panel) during a face-to-face meeting. The coding panel will first determine the feasibility of measuring candidate indicators and risk-adjustment factors using administrative health data, and, second, select the diagnosis, procedure, and drug codes to be included in and excluded from their calculation.

#### Empirical Measurement

We will empirically measure crude and risk-adjusted GPSIs retained at the end of the second stage at the hospital, territorial, and national levels for the years 2012 to 2014 using Swiss and French data. We will conduct sensitivity analyses to assess the impact of different definitions and selected codes on the robustness of empirical results. This stage will also aim to explore the consistency of the empirical results with the literature, highlight variations in indicators across hospitals or health territories, and examine potential bias related to insufficient adjustment on patient case mix or variation in coding practices. We will exclude GPSIs not performing well from the validation stage.

#### Validation of GPSIs

We will test the reliability and validity of retained GPSIs using a comprehensive validation framework. (1) *Face and content validity assessment*: the apparent and content relevance of GPSIs will be discussed by the clinical and coding panels. (2) *Criterion validity assessment*: we will assess the algorithm accuracy of each retained GPSI by measuring its performance in identifying the corresponding adverse event compared with a reference standard (medical record screening). GPSIs with low sensitivity (<75%) or low positive predictive value (PPV) (<75%) will be excluded from the candidate list [50]. We will similarly assess the accuracy of the algorithm developed for each case-mix factor based on coded administrative health data. (3) *Construct validity assessment*: we will verify statistical associations among unadjusted GPSIs, between unadjusted GPSIs and related process indicators, and between unadjusted GPSIs and other unadjusted outcome indicators assessing the same care processes (eg, mortality rates, length of stay, and potentially avoidable readmissions rates). GPSIs with nonsignificant or inconsistent correlations will be excluded from further development and validation processes. (4) *Predictive validity assessment*: finally, for each remaining GPSI, we will assess the statistical performance of its risk-adjustment model in accounting for actual differences in case mix, and therefore in predicting the related outcome, by measures of its calibration and discriminatory power.

#### Analyses of GPSI Geographic and Temporal Variations

(1) We will analyze *geographic variations* at the hospital and territorial levels using funnel plots to identify outliers. Statistical modeling will identify potential causes of systematic variations related to the health system or the quality of care. (2) We will study *temporal variations* in monthly or quarterly measures of GPSIs for the period 2012-2014 at the national and territorial levels using statistical models to identify potential trends and explanatory factors. We will also study temporal variations at the hospital level, hospital legal status level (ie, public, private not-for-profit, and private for-profit), and hospital volume level (ie, tertiles or quintiles of annual index hospital stays eligible for GPSI denominator) using control charts to identify special causes of variation related to the quality of the health care processes.

#### Data Visualization

For both Switzerland and France, we will construct comparative graphic displays of the anonymized results for scientific, public, or institutional reporting. They will comprise (1) a Swiss and French atlas documenting territorial variations in risk-adjusted GPSIs; (2) funnel plots reflecting between-hospital and between-hospital category variations in risk-adjusted GPSIs; (3) individual control charts of risk-adjusted GPSIs displaying temporal variations for each hospital, hospital category, and territory; and (4) national, territorial, and individual temporal trends in GPSIs over the period 2012-2014.

Regarding GQIs, the clinical panel will select candidate indicators among those suggested in Textbox 1. We will then apply a similar development and validation process, except for criterion and predictive validity assessment. Indeed, as anticoagulant treatments (drug, dose, frequency, and duration) coded in insurance claims data reflect quite precisely the ones that were prescribed and reimbursed, GQI algorithms should be accurate for assessing the management of anticoagulants at, and after, discharge. In addition, we will not be able to access outpatient medical records, which would constitute the suitable reference standard for testing the accuracy of GQIs. We will not assess the predictive validity of GQIs because process indicators do not require case-mix adjustment when comparing hospitals or health territories. Indeed, the quality of hospital care processes does not usually depend on inpatient case mix [37,51].

### GPSI and GQI Development

#### Study Design

To develop GPSIs or GQIs, we will conduct multicenter retrospective observational cohort studies based on insurance claims data individually linked to hospital discharge data.

#### Population Setting

In Switzerland, the study population will consist of all residents aged 65 years and over admitted to a Swiss acute care hospital at least once between 2012 and 2014 (ie, the inclusion period) and insured under the compulsory basic health insurance scheme by Helsana Group for at least 1 year before admission and 1 year after discharge. Helsana is one of the 3 biggest insurance groups in Switzerland, covering approximately one-fifth of the Swiss population aged 65 years and over [52].

Inclusion criteria for the French study population will be similar except that they will target older residents admitted to a French acute care hospital and insured under the general scheme by the National Health Insurance Fund for Salaried Workers (Caisse Nationale d’Assurance Maladie des Travailleurs Salariés [CNAMTS]). This scheme covers approximately 69% of the French population aged 65 years and over (2014 data provided by CNAMTS).

We will exclude from both study populations patients for whom (1) 2012-2014 administrative health data were incomplete, (2) individual data linking was not possible, and (3) hospital length of stay was less than 2 days.

#### Candidate GPSIs and GQIs

AHRQ PSIs are measures based on administrative health data that “screen for adverse events that patients experience as a result of exposure to the healthcare system, and that are likely amenable to prevention by changes at the system or provider level” [19]. Likewise, candidate GPSIs will screen Swiss and French linked administrative data for thromboembolic or hemorrhagic adverse events that resulted from exposure to medical or surgical conditions requiring anticoagulant treatments, and occurred during hospital stay or after discharge (Textbox 1). Hospital-level GPSIs will measure, for each acute care hospital, the cumulative incidences of in-hospital and postdischarge anticoagulant-related adverse events and be defined with a denominator (ie, index stays in a given hospital within a 1-year period) and a numerator (ie, denominator stays resulting in the adverse event of interest during hospitalization and up to 30 days, 60 days, 90 days, 6 months, and 1 year after discharge). For example, the denominator of the GPSI that will measure the 2012 cumulative incidence of in-hospital and 30-day postdischarge hemorrhagic events for older patients receiving venous thromboprophylaxis after elective total hip arthroplasty will include any 2012 hospital stay for elective total hip arthroplasty, excluding 1-day surgery, of patients aged 65 years and over. Indeed, older patients undergoing elective total hip arthroplasty are supposed to receive the appropriate venous thromboprophylaxis, as they are considered to be at high risk of thromboembolic and hemorrhagic events [53]. The numerator will comprise any hemorrhage [54] occurring during index hospital stays and up to 30 days after the patients’ discharge dates. The hospital that performed the total hip arthroplasty procedure will be held accountable for the index stay and hemorrhage. We will identify index stays and hemorrhages using hospital discharge data and linked administrative health data, respectively.

For each GPSI, 2 GQIs will measure for each hospital (1) the proportion of index hospital stays resulting in the recommended anticoagulation (drug, dose, frequency, and duration) at discharge, and up to 30 days, 60 days, 90 days, 6 months, and 1 year after discharge; and (2) the median duration of recommended anticoagulant treatment after discharge for index hospital stays. For example, the GPSI described above will be completed by the following GQIs: (1) the proportion of 2012 hospital stays for elective total hip arthroplasty in patients aged 65 years and over resulting in the recommended anticoagulation (drug, dose, frequency, and duration) at and up to 30 days after discharge; and (2) the median duration of recommended anticoagulant treatment after discharge for these index stays. We will extract information on anticoagulant treatments after hospital discharge from insurance claims data.

#### Administrative Health Data Scope and Time Frame

We will extract Swiss administrative health data from the Helsana Group data warehouse and will include 2010-2015 insurance claims data individually linked to 2012-2015 acute care hospital discharge data and individual measures of dependency for nursing home residents. Since data collection of inpatient diagnosis and procedure codes and dependency measures started in 2012, only insurance claims data will be available for the period 2010-2012.

We will extract French data from the national health insurance information system (Système National d’Information Inter-Régimes de l’Assurance Maladie [SNIIR-AM]) hosted by CNAMTS [55]. Since 2007, SNIIR-AM has included individually linked data from various administrative databases for the entire French population: all hospital discharge data (ie, discharge data from acute, postacute, rehabilitation, psychiatric, and long-term care hospitals and from hospital-at-home facilities); insurance claims data; data on nursing homes residents; and data on health professionals’ characteristics. We will include only those patients insured under the general scheme, as vital status and death date are exhaustive only for these patients.

Multimedia Appendix 1 comprehensively describes Swiss and French data, including linkage methods and success rates.

In both countries, we will identify index hospital stays and in-hospital adverse events from 2012-2014 hospital data and adverse events up to 1 year after discharge from 2012-2015 linked hospital and insurance data (Textbox 2). Similarly, we will test anticoagulation management up to 1 year after discharge over the period 2012-2015 using linked data (Textbox 2). GPSI risk-adjustment factors will be estimated using data from 2010-2014 to account for patient conditions up to 2 years before their admission. Since hospital discharge data will not be available for the period 2010-2012 in Switzerland, we will derive risk-adjustment factors from insurance claims data only (Textbox 2).

**Geriatric patient safety indicator (GPSI) and geriatric quality indicator (GQI) measurement time frames.**

**GPSIs**

Inclusion period for index hospital stay (denominator)

- Discharge date between January 1, 2012 and December 31, 2014

Follow-up period for adverse event screening (numerator)

- From admission date of index hospital stay up to 1 year after discharge date of index hospital stay

Inclusion period for case-mix factors present at admission (risk adjusters)

- Up to 2 years before admission date of index hospital stay

**GQIs**

1. Proportion of index hospital stays resulting in the recommended anticoagulation at and after discharge

Inclusion period for index hospital stay (denominator)

- Discharge date between January 1, 2012 and December 31, 2014

Follow-up period for prescription of anticoagulant treatment (numerator)

- Up to 1 year after discharge date of index hospital stay

2. Median duration of anticoagulant treatment after discharge for index hospital stays

Follow-up period for prescription of anticoagulant treatment

- Up to 1 year after discharge date of index hospital stay

### GPSI Criterion Validity Assessment

#### Study Designs

The study design considered to assess the criterion validity of each GPSI will be a multicenter cross-sectional study with a test-based enrollment approach [56].

#### Population Settings

For each GPSI, the study population will comprise all older insured patients who were admitted to an acute care hospital between 2013 and 2014 and were at risk for the related adverse event (GPSI denominator population). Whereas any acute care hospital may be included in the Swiss validation study, the French study will target acute care hospitals located in the Burgundy-Franche‑Comté region, which hosts Dijon University Hospital (ie, the French collaborative research center). Indeed, as these studies are time and resource consuming, it is necessary to balance representativeness against efficiency. For both countries, exclusion criteria will include an insufficient number of at-risk admissions over the 2 years 2013 and 2014 (≤50 stays) and a hospital’s refusal to participate.

#### Data Source and Criterion Validity Metrics

We will assess GPSI accuracy by measuring the performance of the algorithm in identifying corresponding adverse events based on administrative health data compared with a reference standard (medical record screening). We will identify GPSI+ (ie, complicated) and GPSI– (ie, uncomplicated) hospital stays from administrative data based on the algorithm. Then, we will randomly select a sample of these GPSI+ and GPSI– stays and verify whether an adverse event is recorded in the corresponding medical record. GPSI criterion validity will then be assessed based on the sensitivity, specificity, PPV, and negative predictive value of the algorithm.

### Planned Statistical Analyses and Sample Size Calculation

Statistical analyses will be performed using Stata/MP software version 14 for Windows (StataCorp LLC) or SAS/STAT software, version 9.4 of the SAS system for Windows (SAS Institute Inc).

#### Indicator Calculation and Case-Mix Adjustment

GPSIs will provide yearly (2012-2014) observed cumulative incidences of thromboembolic and bleeding adverse events in selected surgical procedures or medical conditions (Textbox 1) for each hospital, hospital legal status, hospital volume category, health territory (ie, Swiss cantons and French departments), and country. We will calculate these incidences as the proportion of at-risk hospital stays resulting in in-hospital or postdischarge adverse events over a year. To allow comparisons across hospitals and health territories, we will adjust GPSIs on patient case mix (eg, age, sex, multimorbidity, frailty, disability, polypharmacy, point of origin for admission, admission mode, thromboembolic or hemorrhagic individual score, and local health care capacity) using multilevel logistic regression modeling accounting for the hierarchical structure of the data. Every GPSI risk-adjustment model will undergo a 3-fold cross-validation, in which we will use a random sample comprising one-third of the whole data to develop the empirical model (development dataset), another one-third to estimate the parameters (estimation dataset), and the remaining one-third to test the predictive validity of the model (validation dataset) [57]. The predictive validity of the model will be assessed by measures of its calibration (Hosmer-Lemeshow goodness-of-fit test) and discriminatory power (C statistic) [58]. We will provide expected cumulative incidences with their 95% confidence intervals.

We will calculate GQIs as (1) the proportion of at-risk hospital stays resulting in the recommended anticoagulation (drug, dose, frequency, and duration) at discharge and up to 30 days, 60 days, 90 days, 6 months, and 1 year after discharge; and (2) the median duration of recommended anticoagulant treatment after discharge for index hospital stays. Anticoagulation management for selected at-risk conditions (Textbox 1) will be assessed at the hospital, hospital legal status, hospital volume category, territorial, and national levels and for each year of the inclusion period. Process indicators will not be risk adjusted.

#### Case-Mix Factor Calculation

Case-mix factors usually considered for PSI risk adjustment include age, sex, past medical history, point of origin for admission, admission mode, and comorbidity present at admission [19,36]. We will also consider other important predictors of adverse health outcomes, including thromboembolic or hemorrhagic events, in older inpatients. To this end, multimorbidity, polypharmacy, frailty, and disability indexes based on Swiss and French administrative health data will be purposely developed or adapted from already validated ones [38-40,59-62]. Although these conditions are considered as separate clinical entities and independently associated with poor outcomes, they overlap significantly and are causally interrelated [4,41,61,63]. Consequently, risk-adjustment models will also account for possible interactions or associations between these various case-mix variables. We will also consider a single proxy measure of older inpatient complexity—similar to the Charlson and Elixhauser indexes—that could encompass all these conditions. GPSIs will also be adjusted on individual thromboembolic or bleeding risk scores, including venous thromboembolism risk scores for surgical patients (Caprini and Rogers scores) [44], ischemic stroke risk scores for patients with atrial fibrillation (CHA_2_ DS_2_-VASc or ATRIA score) [43], and a major bleeding risk score for patients on anticoagulation (HAS-BLED) [43]. CHA_2_ DS_2_-VASc and HAS-BLED have already been estimated based on French data [45] and will be adapted to Swiss data. We will develop the remaining scores in both countries.

#### Studies of GPSI and GQI Geographic and Temporal Variations

We will study variations across hospitals, hospital legal status, hospital volume categories, and health territories using (1) funnel plots to identify potential outliers [64-66]; (2) hierarchical logistic regression models to identify potential causes of systemic variations related to the health system (eg, health care supply, including specialized geriatric units or professionals in hospitals; availability of integrated care organizations; health policy strategy supporting patient safety) or to the quality of care (eg, availability of professional guidelines, adherence to treatment and surveillance standards regarding anticoagulation); and (3) propensity score-based risk-adjustment models to estimate the performance of the different hospitals assessed by validated GPSIs or GQIs [67].

We will study monthly or quarterly variations in GPSIs and GQIs over the inclusion period at the national and territorial levels using multilevel logistic regression modeling for repeated measures to identify potential trends and explanatory factors (eg, changes in anticoagulation guidelines, coding rules, or classifications, Diagnosis-Related Group (DRG) system, or drug and procedure reimbursement limits). We will also assess temporal variations at the hospital, hospital legal status, and hospital volume levels using control charts, including p-charts and cumulative sum charts [68-71], to identify special causes of variation related to the quality of health care processes.

#### Comparisons of Anticoagulant-Related Safety Between Switzerland and France

We will compare GPSIs measured at the national level between Switzerland and France using direct age and sex standardization, as recommended by the Organisation for Economic Co-operation and Development [16]. Indeed, age and sex standardization enable accounting for between-country differences in population structures, including general and inpatient populations, and practices regarding older inpatients’ hospitalization in acute care [21]. For each GPSI, aggregated nationwide counts of index hospital stays stratified by age and sex will define the internal reference population. We will then calculate a comparative morbidity figure (ie, the ratio of directly age- and sex-standardized adverse event rates in Switzerland and France) along with its 95% confidence interval. Moreover, to account for differences in coding systems, we plan to adjust national measures of GPSIs on the mean number of secondary diagnoses among denominator cases [21].

## Results

### Administrative Health Data and Study Populations

After accounting for inclusion and exclusion criteria, we will include 166,670 Swiss and 5,902,037 French residents aged 65 years and over in our study. Multimedia Appendix 1 comprehensively describes Swiss and French data available for our study, along with data linkage methods and success rates.

### Sample Size Calculation for GPSI Criterion Validity Assessment

For sample size calculation, we used a test result-based sampling method [72] to minimize the number of medical records to be abstracted. We estimated optimal sample sizes, as well as sampling fractions of GPSI+ and GPSI– medical records to be abstracted, for prevalence (ie, proportion of medical records in which at least 1 adverse event was recorded) ranging from 0.4% to 5%, a desired algorithm sensitivity of 75%, a 20% width for sensitivity 95% confidence interval, and unknown numbers of at-risk stays (ie, unknown GPSI denominators). We extracted prevalence values and PPVs from the literature [16,24,32-34,44,50,73-75] and calculated sample size using Stata/IC software version 13 (see Multimedia Appendix 2). Thus, assuming a prevalence of 2%, an optimal sample of 3164 medical records should be randomly selected from participating hospitals and abstracted: 126 GPSI+ and 3038 GPSI– medical records. These results are consistent with the literature [73].

### Ethical and Regulatory Aspects

The research project was approved by the Cantonal Ethics Commission of Vaud (Switzerland) in August 2016 (Decision CER-VD 2016-00508) and by the French data protection authority (Commission National de l’Informatique et des Libertés) in March 2016 (Decision DE-2016-036).

### Timetable of the Project

All administrative health data are now available to the Swiss and French research teams. The research project will run until the end of 2018.

## Discussion

### Main Strengths of the Study

First, to our knowledge, this is the first study aiming at developing and validating a set of geriatric quality and safety indicators based on linked administrative health data, both in Switzerland and in France. Assessing the quality and safety of anticoagulation in older inpatients based on such data is also innovative. Indeed, in addition to using extended PSI-09 and PSI-12, which capture perioperative adverse events up to 30 days after discharge [22], we will develop and validate a set of GPSIs assessing thromboembolic or bleeding adverse events for various surgical procedures or medical conditions requiring curative or prophylactic anticoagulation. Similarly, our set of validated GQIs will complement the ACOVE-3 quality indicators based on administrative health data assessing warfarin prescription and surveillance in older patients with heart failure or atrial fibrillation [23]. In particular, they should provide useful information on direct oral anticoagulant management.

Second, validated GPSIs will account for an older inpatient case mix, which is crucial when comparing hospitals or health territories. Specific indexes based on Swiss and French administrative health data, including multimorbidity, polypharmacy, frailty, and disability indexes, will thus be purposefully developed or adapted from existing ones to target older inpatients’ conditions at admission [38-40,59-62]. These important predictors of adverse health outcomes (ie, mortality, morbidity, dependency, and institutionalization), health-related quality of life, and resource use [38,40,59] may be used not only as risk-adjustment factors for other health care quality and safety indicators, but also as screening tools for older patients’ vulnerability in various health care settings. Indeed, many countries, including France, try to implement frailty or vulnerability screening and management programs (eg, the French initiative “parcours de santé pour les personnes âgées en risque de perte d’autonomie” [PAERPA]) to avoid institutionalization and hospitalization, improve older citizens’ quality of life, and reduce health care costs [76,77]. Finally, assessing patient complexity and vulnerability, both at the individual and population levels, may contribute to better planning of appropriate health and social care services [76,77].

Developing or adapting individual thromboembolic or bleeding risk scores based on administrative health data is the third major strength of our study. These scores will be included in GPSI risk-adjustment models and may also contribute to assessing the quality of anticoagulant management by comparing patients’ risks with the treatment they actually received. Indeed, decisions regarding anticoagulant treatments (ie, type, dose, frequency, and duration) and surveillance should be consistent with risk-benefit assessment, especially in the older inpatient population, which is highly exposed to anticoagulant-related thromboembolic or bleeding complications. Furthermore, complementing individual scores with patient outcomes (ie, GPSIs) and data on anticoagulant management (ie, GQIs) should enrich information on effectiveness and safety of anticoagulants in “real life,” in particular direct oral anticoagulants, and eventually help improve professional guidelines [25,26,30,31].

Fourth, our project will enable comparisons of GPSIs and GQIs between Switzerland and France, which is a source of cross-country learning and performance improvement [17,18]. Indeed, despite having different organization, governance, and financing, and serving different populations, Swiss and French health systems “have similar goals and face similar challenges, such as demographic change, limited resources and rising costs” [17,18]. Between-country comparisons should thus help study differences in (1) linked administrative health data features, quality, regulation, and coding rules; (2) older inpatients’ and health care providers’ characteristics; (3) quality standards on and safety of anticoagulant management in older inpatients; and (4) health care policy and reforms toward transparency, accountability, and high-quality safe care to vulnerable older inpatients [12,15].

### Study Limitations

Our study has several limitations that should be considered when interpreting the results. First, our study populations may not be representative of all Swiss and French older inpatients. Indeed, Swiss data cover approximately 18% of the Swiss population aged 65 years and over [52], and policy holders residing in German-speaking cantons are overrepresented compared with others. French data include a very large population of nearly 6 million persons, namely 69% of the French insured aged 65 years and over. However, only salaried or retired employees are represented, while other professional subgroups and enrollees in 1 of the 16 specific insurance schemes (eg, soldiers, miners, ministers of religion, and employees of the French National Railway Company) are excluded from the study population [55]. As insured or cultural groups may differ in their risk factors, compliance with anticoagulant treatments, access to and utilization of health services, and geographic distribution (eg, employees may be underrepresented in rural areas), the internal and external validity of our study might be affected by selection bias [78]. Albeit figuring that this bias should not significantly affect our indicators, we will thus generalize our findings to the study populations and gather information on older inpatients excluded from our study. In particular, we will make a request to CNAMTS to access their data on French residents enrolled in other schemes than the general one. French data will thus cover the entire population.

Second, insurance claims data may also be incomplete or “selected.” For example, they will not include coded diagnoses on adverse events occurring in outpatient settings and will not provide information on patients’ adherence to treatment and surveillance. However, we expect to capture almost all postdischarge adverse events by screening readmissions, prescriptions or modifications of anticoagulant or antihemorrhagic treatments, outpatient procedures (eg, lower limb venous ultrasonography), and laboratory tests (eg, hemoglobin and international normalized ratio tests). Moreover, we believe that the quality of anticoagulant management is accurately assessed using large administrative databases linked over time. Swiss insurance claims may also be missing differentially according to health insurance deductibles (ie, SwF 300 to 2500 per year) [78-80]. Indeed, policyholders with the highest deductibles tend not to claim reimbursement of medical expenses when their annual amount does not reach the deductible, which leads to missing claims. However, this potential selection bias should not be significant, since health insurers estimate that only 2% to 3% of all invoices are not sent for reimbursement [52]. Finally, French data from SNIIR-AM may also lack some information regarding fully reimbursed long-term conditions. Indeed, long-term conditions are often underreported in patients fully covered for several long-term conditions or benefiting from a complementary insurance, or in nursing home residents [81]. We will overcome this limitation by deducing missing long-term conditions from medications coded in insurance claims or from diagnoses coded in hospital discharge records.

Third, hospital discharge data may also provide incomplete information. For example, data on inpatient medications will be missing for both countries, as they are not mandatory for reimbursement. We will then infer the prescribed anticoagulant treatment during hospital stay from that prescribed just after discharge using insurance claims data. Also, in Switzerland, we will not be able to identify reasons for readmission or retransfer to the same hospital within 18 days after discharge if the second stay is grouped in the same Major Diagnostic Category as the initial one. Indeed, according to the SwissDRG billing rules, such a readmission or retransfer is merged with the initial admission, leading to a single stay and discharge record. Furthermore, Swiss hospital discharge data are limited to acute care hospitals and do not cover hospital-at-home facilities, and rehabilitation or psychiatric hospitals. Thromboembolic and hemorrhagic adverse events occurring in these settings will thus be missed except severe ones that would necessitate transfer to acute care facilities.

Fourth, in both countries, neither hospital discharge data nor insurance claims data contain individual information on causes of death—which could be related to thromboembolic and hemorrhagic adverse events—or on significant factors that should be accounted for in GPSI case-mix adjustment. These factors include, for example, genetic factors, demographic characteristics (eg, ethnicity), clinical factors (eg, vital signs, results from clinical examinations or laboratory tests), health-related behaviors (eg, excessive alcohol consumption, diet, and physical activity), health literacy, and patient preferences or cultural beliefs [36].

Fifth, hospital discharge data may have potential limitations regarding coding accuracy. In particular, the quality of diagnostic and procedure coding may be affected by the quality of patient record documentation, coders’ background, training, and experience (eg, clinicians vs professional coders), coding quality controls, and unintentional and intentional coding errors (ie, “gaming” or “upcoding” to increase reimbursement) [36,37,82,83]. Similarly, coding rules and classifications, coders’ characteristics, DRG classification systems, and coding quality assurance policies (eg, coding quality controls, incentives for coding, and penalties for inappropriate coding) may vary significantly among health systems [21]. For example, Swiss and French health systems differ on coding rules for the “principal diagnosis” (ie, “condition responsible for resource use” vs “reason for admission”) [84], mean numbers of secondary diagnoses coded [21], medical coding classifications for diagnoses (*International Classification of Diseases, 10th Revision [ICD-10], German Modification* vs *ICD-10 France*) and procedures (Swiss operation classification [CHOP] vs French shared classification of medical procedures [CCAM]), DRG classification systems (SwissDRG vs French Groupes Homogènes de Malades), and coders’ profiles (professional coders vs mixed profiles, including professional coders and physicians). Limitations in coding quality may introduce systematic bias in GPSI estimates and in comparisons among hospitals, health territories, or countries, which cannot be accounted for by risk adjustment [21,36,37,84,85]. Criterion validity assessment will thus be essential to select valid GPSIs. Moreover, regarding the possible influence of coding practice to increase reimbursement, serious controls and financial penalties are in place in Switzerland to limit this issue. Similarly, serious controls are carried out in France, both at the hospital and at the Technical Agency for Information on Hospital Care (Agence Technique de l’Information sur l’Hospitalisation) levels.

Sixth, we have decided to compare the quality of anticoagulant prescription and surveillance across hospitals without adjusting GQIs for differences in case mix. However, process indicators may require case-mix adjustment when inpatients eligible to receive the related process are not perfectly specified [51] or when the “opportunity for violation of the standards” varies by case mix [37]. Indeed, guidelines related to preventive and curative anticoagulation do not account for older inpatients’ heterogeneity regarding their functional, cognitive, or social disability, health conditions, and complexity. We will thus verify that these conditions have no impact on GQI variations across hospitals and health territories.

Seventh, criterion validity assessment based on retrospective review of medical records may be less methodologically robust than assessing GPSIs based on prospectively collected data [86]. Indeed, many adverse events are not recognized during the process of clinical care without dedicated assessment, and they are thus incompletely captured in medical records [87-90]. However, major adverse events will probably be well reported in medical records, as they will contribute to the use of additional resources in the hospital, and need to be reported for appropriate reimbursement. Moreover, Klopotowska et al showed that “[adverse drug events] with evident causality and with clinically apparent and severe consequences,” such as adverse drug events resulting in hemorrhage or raised international normalized ratio, are well recognized and documented by medical teams [89]. In addition, the implementation of more valid, but more resource-intensive, studies would require important financial support that will not be easy to obtain, neither from research agencies nor from health care services.

Eighth, prior validation studies have suggested that, despite good predictive and construct validity, AHRQ PSIs demonstrate moderate sensitivity and PPVs in detecting surgical adverse events [22]. Indeed, PSI algorithms usually favor specificity over sensitivity and PPV [91]. Consequently, unless AHRQ PSI validity is improved, more robust adverse event detection methods (eg, prospective monitoring or voluntary patient safety event reporting) should be preferred for internal quality improvement, performance assessment, public reporting, and, above all, pay for performance [83,91-93]. However, recent validation studies contradict these results, at least for PSI-12 and PSI-09 [33,83,91]. Indeed, Mull et al showed that the sensitivity of a PSI-12 algorithm based on *ICD, 9th Revision, Clinical Modification* (*ICD-9-CM*) diagnosis and procedure codes was 65% (95% CI 63%-67%). He also suggested that PSI-12 sensitivity could be improved by using *ICD-10-CM* codes [91]. Similarly, Utter et al reported that the sensitivity of a PSI-09 algorithm based on *ICD-9-CM* codes could reach 85% (95% CI 67%-94%) [33]. Regarding PPVs, Winters et al found, based on a literature review and meta-analysis, pooled PPVs of 63.5% (95% CI 44.3%-82.7%) for PSI-12 and 78.6% (95% CI 73.2%-84.1%) for PSI-09 [83]. In our study, the detection of anticoagulant-related adverse events should also be improved by using hospital discharge data linked to insurance claims data. Indeed, by adapting PSI-12 and PSI-09 algorithms to linked data, Mull et al were able to capture 72% (with PSI-12) and 77% (with PSI-09) additional events occurring up to 30 days after discharge [22]. Finally, in Switzerland, this research project will be integrated into a larger research program, which will aim to develop measures of anticoagulant-related thromboembolic and hemorrhagic adverse events based on structured (ie, administrative and clinical data) and textual data. These measures should, in the end, have better sensitivity and specificity than AHRQ PSIs. The larger research program has just been funded by the Swiss National Fund.

### Conclusions and Perspectives

This innovative study, which is part of a larger research program aiming to develop and validate GPSIs and GQIs in both hospital and ambulatory care settings, will provide valid and reliable outcome and process indicators to assess older inpatient safety related to anticoagulants. It should also provide new information on real-life prevention and treatment of thromboembolism, direct oral anticoagulant prescription and monitoring, and hemorrhagic adverse events related to direct oral anticoagulants in older patients. It should additionally contribute to describing older inpatients’ characteristics and health professionals’ practices in Swiss and French hospital and ambulatory care settings, and help identify geographic and temporal variations in older patient safety related to the health system. Moreover, within the frameworks of the Swiss Health 2020 policy agenda and the French National Health Strategy, the comparative assessment of hospitals and health territories using validated GPSIs and GQIs could help inform health policies, promote transparency, and improve health care efficiency.

## Appendix

Multimedia Appendix 1 Detailed description of Swiss and French health administrative data used in the research project.

Multimedia Appendix 2 Optimal sample size according to proportions of adverse events based on medical record and administrative health data screenings.

## Abbreviations

ACOVE-3: Assessing Care of Vulnerable Elders-3
AHRQ: Agency for Healthcare Research and Quality
CCAM: classification commune des actes médicaux (French shared classification of medical procedures)
CHOP: Swiss operation classification
CNAMTS: Caisse Nationale d’Assurance Maladie des Travailleurs Salariés
DRG: Diagnosis-Related Group
GPSI: geriatric patient safety indicator
GQI: geriatric quality indicator
ICD-9-CM: International Classification of Diseases, 9th Revision, Clinical Modification
ICD-10: International Classification of Diseases, 10th Revision
PAERPA: Parcours de santé pour les personnes âgées en risque de perte d’autonomie (French Healthcare Pathway for frail elderly people)
PPV: positive predictive value
PSI: Patient Safety Indicator
SNIIR-AM: Système National d’Information Inter-Régimes de l’Assurance Maladie
