# Supplementary material for: Geriatric Patient Safety Indicators Based on Linked Administrative Health Data to Assess Anticoagulant-Related Thromboembolic and Hemorrhagic Adverse Events in Older Inpatients: A Study Proposal
Source: JMIR Res Protoc. 2017 May 11;6(5):e82. doi: 10.2196/resprot.7562 (PMC5445236; doi:10.2196/resprot.7562)
Supplement: Multimedia Appendix 1 [file resprot_v6i5e82_app1.pdf]

Swiss health administrative data

Swiss data will be extracted from the Helsana insurance group data wharehouse and comprise individual information on patients aged 65+ admitted in a Swiss acute care hospital at least once between 2012 and 2014, and insured under the compulsory basic health insurance scheme by the Helsana group for at least one year before admission and one year after discharge. Individual data on the Swiss population study for the period 2010-2015 include (Figure 1):

- Insurance claims data extracted from patient invoices sent to the insurance for reimbursement:
  - Information on patient characteristics and insurance coverage: age at first index admission, sex, birth and death date, vital status at the end of the observation period, postal code, national language area (latin vs german language), begin and end date (month, year) of coverage by the basic health insurance scheme, premium region and canton applied, deductible class selected, insurance model chosen, choice of a managed care model, choice of a private or semi-private hospitalization complementary insurance coverage, choice of additional accident coverage;
  - Information on patient chronic morbidity at first index admission: chronic conditions among a list of 22 ones identified from computerized algorithms based on the World Health Organization Anatomical Therapeutic Chemical (ATC) classification system and relating pharmacy-based cost groups (PCGs) to chronic conditions, updated Chronic Disease Score (CDS) [1];

- Information on the category of treatment reimbursed (ambulatory, stationary, home care, daily allowance, others) and insurance claim (disease, accident, motherhood, pension, others);
- Information on ambulatory care services prescribed, dispensed in medical practices and hospitals, and reimbursed to outpatients by the basic health insurance: type of medical and paramedical service (e.g. consultation, visit, transport, nursing care, physiotherapy, occupational therapy, psychotherapy), service duration, place of medical service (ie outpatient practice or hospital department), type of physician contact (face-to-face, phone, home visit), medication codes (Pharmacode from Swissmedic[a], ATC codes, medication active ingredients derived from Swissmedic Index Therapeuticus, product names), imaging (TARMED[b] code and description), laboratory tests (TARMED code and description), medical devices, dates of prescription and dispensing for services, medications, laboratory tests, imaging, and medical devices;
- Prescribers' characteristics in outpatient settings: physician and other healthcare professionals' specialities, canton and postal code of practice;

- Acute care hospital discharge data collected by general care hospitals, surgery and geriatrics specialized clinics and reimbursed by the insurance: Medical diagnoses coded according to the International Classification of Diseases, Tenth Revision, German Modification (ICD-10-GM) versions 2008 and 2010, surgical procedures performed during hospital stay coded according to the Swiss classification for surgical procedures

[a] Swissmedic is the Swiss agency for the authorization and supervision of medicinal products and medical devices (https://www.swissmedic.ch).
[b] TARMED is a standard tariff that applies for all medical and paramedical services provided in medical practices and hospitals in every Swiss canton.

(CHOP), diagnosis-related groups (Swiss-DRG) in which the stays were grouped, admission and discharge dates, and length of stay;

- Information on nursing home residents: admission and discharge dates, dependency scores based on the Resident Assessment Instrument (RAI) [2] and the computerized planning tool of required nursing (outil de PLAnification Informatisée des Soins Infirmiers Requis (PLAISIR) [3];
- Aggregated costs for each hospital or nursing home stay and ambulatory care episode.

Individual administrative health data collected by Helsana health insurance for billing purposes (i.e. insurance claims data, hospital discharge data, and information on nursing home residents) contain identifying variables. They are anonymised using an insured anonymized identifier (random number generated for each insured) before being transferred to Helsana's data warehouse and linked across sources at the individual level. Data scientists from Helsana's Department of Health Sciences only access the anonymised linked database and extract part of it for research projects, including this one. Although data linking between data sources is exhaustive, some data may be missing as Swiss health insurers estimate that 2% to 3% of all invoices are not sent for reimbursement.

Additional information on socio-economic and education levels, spoken languages, older people density and healthcare supply of the patient MedStat region of residency[c] will be secondarily linked to extracted data. They will be provided by a commercial geographic information system (GIS) company (MicroGIS SA, St-Sulpice, Switzerland) and linked to the Helsana database using the MedStat region code.

---

[c] MedStat is a classification of 705 Swiss geographical regions which are aggregated zip codes areas. They were created by the Swiss Federal Office of Statistics to account for demographic, socioeconomic and geographic criteria in geographical comparisons. The classification is available at: http://www.bfs.admin.ch/bfs/portal/fr/index/infothek/nomenklaturen/blank/blank/medstat/02.html)

French health administrative data

French data will be extracted from the national health insurance information system (Système National d'Information Inter-régimes de l'Assurance Maladie, SNIIR-AM) hosted by the National Health Insurance (Caisse nationale de l'assurance maladie des travailleurs salariés, CNAMTS). They comprise individual information on patients aged 65+ admitted in a French acute care hospital at least once between 2012 and 2014, and insured under the general scheme by CNAMTS for at least one year before admission and one year after discharge. Individual data on the French population study for the period 2010-2015 will include (Figure 1):

- Insurance claims data collected by local (public) health insurance centres:
  - Information on patient characteristics and insurance coverage: birth and death dates, sex, community of residence, department and region of residence, community of residence deprivation index developed by Rey et al [4];
  - Information on the patient chronic morbidity: medical diagnoses coded according to the International Classification of Diseases, Tenth Revision, French Version (ICD-10-FR) for patients affected by at least one of the 30 long-term conditions (LTCs) allowing for co-payment exemption (Affection de Longue Durée, ALD); frequent, severe, progressive or costly conditions or treatments within a list of 56 ones identified from computerized algorithms - also known as the "mapping tool" - mainly based on ALD and medication ATC codes [5];
  - Information on ambulatory care services dispensed in medical practices and hospitals and reimbursed to outpatients by the National Health Insurance: type of medical and paramedical services (e.g. visits to primary care physicians or specialists, transport, physiotherapy, nursing care, occupational therapy, nutrition

counselling, etc); medications delivered by community or hospital pharmacies and coded with the ATC classification system, the French Marketing Authorization presentation classification (Code Identifiant de Présentation, CIP), or the French Common Units of Dispensation classification (UCD codes), diagnostic or treatment procedures performed in primary care practices or outpatient clinics including imaging coded with the French classification for procedures (Classification Commune des Actes Médicaux, CCAM), biological tests coded with the French classification for biological test (Nomenclature des Actes de Biologie Médicale, NABM); medical devices coded with the French list of medical devices (Liste des Produits et Prestations remboursables, LPP), dates of prescription and dispensing for services, medications, laboratory tests, imaging, and medical device;

  - Information on visited ambulatory care professionals (mostly physicians and pharmacists) contained in the national shared directory of health professionals (Répertoire Partagé des Professionnels de Santé, RPPS): age on 1 January of the relevant year, gender, conventional sector, activity, specialty, type of practice, town or different places of practice;

- Hospital discharge data from all public and private acute care hospitals (PMSI-MCO): diagnoses (ICD-10-FR codes) and procedures (CCAM codes) performed during hospital stay, DRGs (Groupe Homogène de Malades, GHM) in which the stays were grouped, costly and highly essential medications within a specific list updated every year, medical devices (LPP codes), movements (provenance, destination, number of inhospital

transitions), admission and discharge modalities (e.g. emergency admission), discharge date and length of stay;

- Hospital discharge data from other hospital settings including rehabilitation (PMSI-SSR), psychiatric (PMSI-PSY), and hospital-at-home settings (PMSI-HAD).
- Information on nursing home residents: movements (admission and discharge dates) and medical expenditures (RESID-EHPAD database).

All administrative health data sources are linked using the pseudonymized national registration number (NIR). Indeed, both insurance claims data and hospital discharge data, respectively collected by local health insurance centres and hospitals, contain identifying variables, such as patient NIR, name, sex or birth date, for all French residents except undocumented immigrants. Before being transferred to the SNIIR-AM, these data are pseudonymized using a non-reversible, two-level, nominative information occultation function (fonction d'occultation d'informations nominatives, FOIN) [6,7]. Local health insurance centres are in charge of pseudonymizing insurance claims data while hospitals and the technical agency for hospital information (Agence Technique de l'Information sur l'Hospitalisation, ATIH) are responsible for pseudonymizing hospital discharge data. Similarly, information on nursing home residents are linked to other SNIIR-AM data sources using the pseudonymized NIR which is generated at the local level (i.e. local health insurance centre). Finally, information on health care professionals are linked to patient data using individual RPPS numbers which are attributed by professional orders and notified on insurance claims. The success rate for linkage between hospital discharge data and insurance claims data is higher than 99% (2016 data provided by CNAMTS). Linkage errors result from miss-recorded birth dates, missing pseudonymized NIR for undocumented immigrants, or patient miss-identification for same-sex twins who have the same pseudonymized NIR.

Figure 1. Swiss and French linked administrative health data scopes and time frames

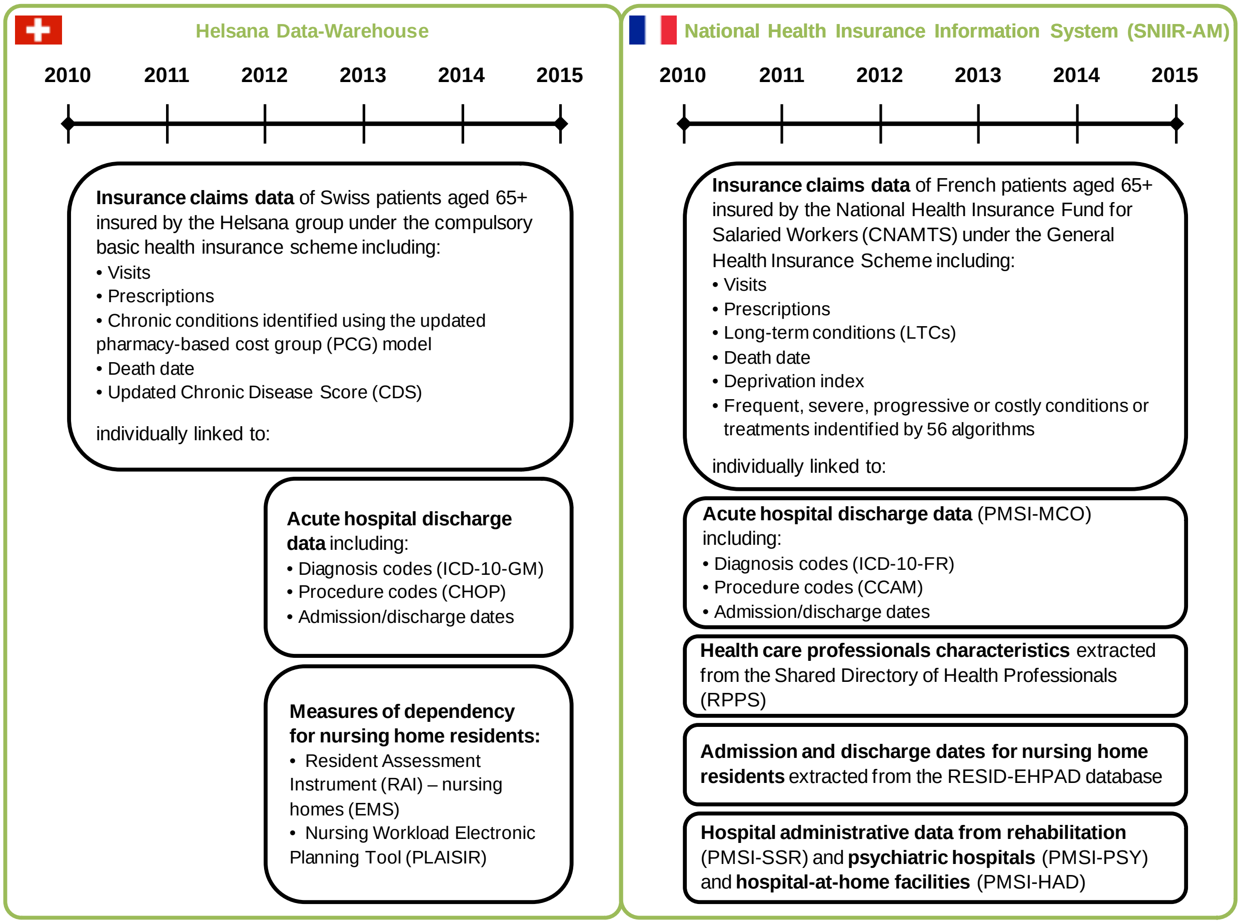

References

1. Huber CA, Schneeweiss S, Signorell A, Reich O. Improved prediction of medical expenditures and health care utilization using an updated chronic disease score and claims data. J Clin Epidemiol. 2013;66(10):1118-27. PMID:23845184

2. Hutchinson AM, Milke DL, Maisey S, Johnson C, Squires JE, Teare G, Estabrooks CA. The Resident Assessment Instrument-Minimum Data Set 2.0 quality indicators: a systematic review. BMC Health Serv Res. 2010;10:166. PMID:20550719

3. Seematter-Bagnoud L, Fustinoni S, Meylan L, Monod S, Junod A, Büla C, Santos-Eggimann BRds, 191). Le Resident Assessment Instrument (RAI) et autres instruments d'évaluation, d'orientation et de communication relatifs aux personnes âgées dans le réseau de soins (Raisons de santé ; 191). Lausanne: Institut universitaire de médecine sociale et préventive (IUMSP); 2012. p. 64.

4. Rey G, Jougla E, Fouillet A, Hemon D. Ecological association between a deprivation index and mortality in France over the period 1997 - 2001: variations with spatial scale, degree of urbanicity, age, gender and cause of death. BMC Public Health. 2009;9:33. PMID:19161613

5. Caisse nationale de l'Assurance Maladie des travailleurs salariés. Améliorer la qualité du système de santé et maîtriser les dépenses: les propositions de l'Assurance Maladie pour 2016. Rapport au ministre chargé de la Sécurité sociale et au Parlement sur l'évolution des charges et des produits de l'Assurance Maladie au titre de 2016 (loi du 13 août 2004). Paris: CNAMTS; 2015 Jul. URL:http://www.ameli.fr/rapport-charges-et-produits-2016/. Archived at http://www.webcitation.org/6pNGzYMZx

6. Trouessin G, Allaert FA. FOIN: a nominative information occultation function. Stud Health Technol Inform. 1997;43 Pt A:196-200. PMID:10179536

7. Quantin C, Fassa M, Coatrieux G, Riandey B, Trouessin G, Allaert FA. [Linking anonymous databases for national and international multicenter epidemiological studies: a cryptographic algorithm]. Rev Epidemiol Sante Publique. 2009;57(1):33-9. PMID:19162419
