# Supplementary material for: Geriatric Patient Safety Indicators Based on Linked Administrative Health Data to Assess Anticoagulant-Related Thromboembolic and Hemorrhagic Adverse Events in Older Inpatients: A Study Proposal
Source: JMIR Res Protoc. 2017 May 11;6(5):e82. doi: 10.2196/resprot.7562 (PMC5445236; doi:10.2196/resprot.7562)
Supplement: Multimedia Appendix 2 [file resprot_v6i5e82_app2.pdf]

Prevalence[a]	GPSI[b]	Optimal sample size[c]	GPSI+[d]	GPSI-[d]	Sensitivity (Se, 95%CI)	Specificity (Sp,95%CI)	Positive Predictive Value (PPV,95%CI)	Negative Predictive Value (NPV,95%CI)
(%)	(%)	(n)	(n)	(n)	(%, [%;%])	(%, [%;%])	(%, [%;%])	(%, [%;%])
0.4	0.3	17783	53	17730	75 [64;84]	100 [100;100]	100 [93;100]	100 [100;100]
	0.4	14505	261	14244	75 [69;80]	100 [100;100]	75 [69;80]	100 [100;100]
	0.5	14484	366	14118	75 [64;84]	100 [100;100]	60 [55;65]	100 [100;100]
0.5	0.4	12285	111	12174	75 [64;84]	100 [100;100]	94 [87;97]	100 [100;100]
	0.5	14227	235	13992	75 [69;80]	100 [100;100]	75 [69;80]	100 [100;100]
	0.6	11684	313	11371	75 [64;84]	100 [100;100]	62 [57;68]	100 [100;100]
0.7	0.6	8663	135	8528	75 [67;81]	100 [100;100]	87 [81;92]	100 [100;100]
	0.7	8493	201	8292	75 [64;84]	100 [100;100]	75 [69;81]	100 [100;100]
	0.8	8469	250	8219	75 [64;84]	100 [100;100]	66 [59;71]	100 [100;100]
1	0.8	6466	83	6383	75 [64;84]	100 [100;100]	94 [86;98]	100 [100;100]
	1	6056	171	5885	75 [64;84]	100 [100;100]	75 [68;81]	100 [100;100]
	2	6140	376	5764	75 [64;84]	99 [99;99]	38 [33;43]	100 [100;100]
	5	6495	708	5787	75 [64;84]	96 [95;96]	15 [12;18]	100 [100;100]
2	1.5	3557	53	3504	75 [64;84]	100 [100;100]	100 [93;100]	99 [99;100]
	2	3164	126	3038	75 [64;84]	99 [99;100]	75 [66;82]	99 [99;100]
	4	3208	274	2934	75 [64;84]	97 [97;98]	38 [32;44]	99 [99;100]
	5	3252	325	2927	75 [64;84]	96 [96;97]	30 [25;35]	99 [99;100]
	8	3359	448	2911	75 [64;84]	93 [93;94]	19 [15;23]	99 [99;100]
4	3	1766	32	1734	75 [64;84]	100 [99;100]	97 [84;100]	99 [98;99]
	4	1670	93	1577	75 [64;84]	99 [98;99]	75 [65;84]	99 [98;99]
	5	1655	129	1526	75 [64;84]	98 [97;98]	60 [51;68]	99 [98;99]
	8	1687	201	1486	75 [64;84]	95 [94;96]	37 [31;34]	99 [98;99]
5	4	1415	40	1375	75 [64;84]	100 [99;100]	95 [83;99]	99 [98;99]
	5	1364	85	1279	75 [64;84]	99 [98;99]	75 [65;84]	99 [98;99]
	8	1356	151	1205	75 [64;84]	96 [94;96]	47 [39;55]	99 [98;99]

a. Proportion of adverse events based on medical record screening

b. Proportion of adverse events based on administrative health data screening

c. Optimized number of medical records to be abstracted

d. Optimized numbers of GPSI+ and GPSI- medical records to be abstracted (sample size = GPSI+ + GPSI-)
